# Directional-Freezing-Assisted In Situ Sol–Gel Strategy to Synthesize High-Strength, Fire-Resistant, and Hydrophobic Wood-Based Composite Aerogels for Thermal Insulation

**DOI:** 10.3390/gels9020170

**Published:** 2023-02-20

**Authors:** Yan Hou, Junyong Chen, Defang Pan, Lu Zhao

**Affiliations:** School of Chemistry and Chemical Engineering, Qilu Normal University, Jinan 250200, China

**Keywords:** wood–silica, aerogels, directional freezing, high-strength, fire-resistant, hydrophobicity

## Abstract

The undesirable inherent natural characteristics of wood, such as low mechanical strength, flammability, and hygroscopicity, limit its potential applications in the thermal insulation industry. Overcoming these disadvantages can greatly expand the application scope of wood. A new attempt at wood modification, the directional-freezing-assisted in situ sol–gel strategy, was used to obtain wood–silica composite aerogels with the unique multi-level ordered porous structure of wood. This method enables silica nanoparticles to successfully replace lignin and facilitates the formation of strong hydrogen bonds between the silica and cellulose molecules. This results in improved mechanical properties for the composite with a density similar to that of natural wood but a mechanical strength that can be up to five times greater. The thermal conductivity coefficient is also reduced to 0.032 W (m·K)^−1^ compared to 0.066 W (m·K)^−1^ for natural wood. This aerogel composite exhibits improved fire resistance and hygroscopicity, with a decomposition temperature increase of approximately 45 °C compared to natural wood. Additionally, the composite demonstrates self-extinguishing behavior, with the structure remaining intact after combustion, and thus enhanced fire resistance. Simultaneously, the enhanced aerogel composite hydrophobicity, with water contact angle of up to 120°, is beneficial to a prominent thermal insulation performance in a high-humidity environment. The successful synthesis of wood-based composite aerogels provides a new and innovative approach for the utilization of wood resources in the thermal insulation industry.

## 1. Introduction

Numerous studies have been conducted to investigate thermal insulation materials with excellent performance to improve energy conservation and reduce emissions. Various thermal insulation materials with exceptional performance suffer from either nonrenewability and safety questions (for example, synthetic polymers [1,2,3]), poor mechanical properties (for example, silica aerogels [4,5]), or complex manufacturing processes and thus high cost (for example, graphene nanomaterials [6]). Wood, on the contrary, is a renewable energy material with low density, high porosity, and low thermal conductivity coefficient; therefore, it outperforms other thermal insulators in terms of biocompatibility and biodegradability. In consequence, wood has broad application prospects in the fields of energy, thermal insulation, and pollutant removal [7,8,9]. Wood is a lignocellulosic material primarily comprising cellulose, hemicellulose, and lignin [10,11] that has several hydrophilic groups that absorb water. When wood is used or stored in a humid environment, hygroscopic deformation and mildew occur easily, restricting the application of wood in heat insulation operations [12].

Wood properties such as water resistance and fire resistance as well as its mechanical properties have been found to significantly improve through the utilization of suitable inorganic nanoparticles such as montmorillonite [13,14,15], SiO_2_ [16], Al_2_O_3_ [17], TiO_2_ [18,19], and other inorganic nanomaterials [20]. SiO_2_ is regarded as the most important wood modifier due to its nontoxicity and eco-friendly properties. Bak et al. [21] reported a method for the dimensional stabilization of wood through the in situ formation of microporous SiO_2_ aerogel using a sol–gel process. Silica aerogel was introduced to improve the dimensional stability and decrease the hygroscopicity of wood. Zhang et al. [22] prepared a low-temperature resistant elastic wood–silica aerogel using the sol–gel method. The prepared aerogels demonstrated excellent elasticity, flexibility, torsion, and elastic recovery. Yan et al. [23] reported an organic and inorganic hybrid strategy to develop a wood–silica aerogel that exhibited excellent hydrophobic (127°), flame-retardant (LOI = 44%), and thermal insulation performances. As an excellent inorganic modifier, silica modification always depends on nanoparticles [21,22] or microspheres [23] filling the wood cell lumen. This process tends to destroy the natural wood’s porous structure. Thus, this approach often leads to problems such as poor compatibility between the modifier and wood, and even shedding of the silica powders or layers, thus leading to an underutilization of the wood’s desirable properties. Hence, assuming that the unique multilevel ordered structure of wood is complete, improving its fire resistance, hydrophobicity, and mechanical properties poses a significant challenge.

**Figure 1:**
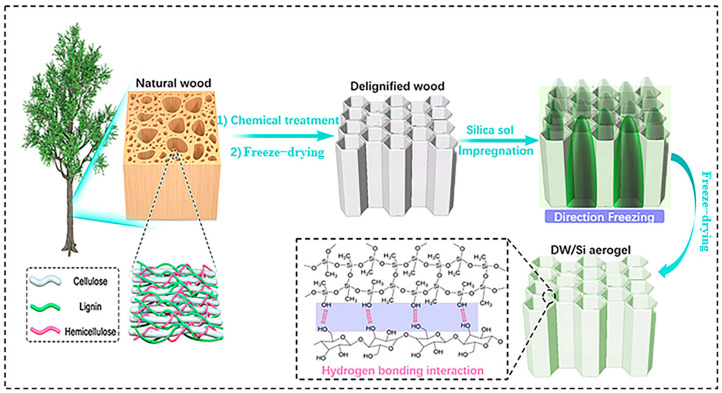
Schematic of the fabrication of wood-based composite aerogels via the directional−freezing−assisted in situ sol−gel strategy.

In this study, a directional-freezing-assisted in situ sol–gel strategy was used to obtain wood–silica composite aerogels with the unique multi-level ordered porous structure of wood. The forming ice crystals during the directional freezing process successfully squeezed silica nanoparticles around and inside the cell wall of wood, enabling silica nanoparticles to successfully replace lignin and achieve a combination with cellulose at the near molecular level, as well as enabling the formation of strong hydrogen bonds between the silica and cellulose molecules, endowing the composite with a series of excellent properties. Using balsa wood as an example, wood-based composite aerogels were prepared (Figure 1). First, a two-step chemical treatment was employed to selectively remove lignin and hemicellulose from the natural wood (NW) in order to retain cellulose with high mechanical strength. The thickness of the cellulose cell wall decreased significantly, thus improving porosity and retaining the orderly arrangement and porous structure of the cellulose nanofibers. Second, the silica sol diffused rapidly into the cellulose cell cavity of the delignified wood (DW) by implementing the immersion method. The composite sample was then placed in a cold trap for directional freezing (the wood axis was perpendicular to the cooling surface). Directional freezing enabled silica nanoparticles to successfully replace lignin and achieve a combination with cellulose at the near-molecular level, then enabled the formation of strong hydrogen bonds between the silica and cellulose molecules, thereby significantly improving the mechanical properties of the wood. Moreover, the thermal conductivity coefficient of the composite was 0.032 W (m·K)^−1^, which was significantly lower than that of NW (0.066 W (m·K)^−1^). The DW/Si-10 aerogel possessed excellent hydrophobicity due to the hydrophobic coating on the cellulose skeleton surface, which, as a result, improved the thermal insulation stability of the wood. To further study the fire resistance properties of the specimen, the modified wood composite was placed in a direct flame from a blowlamp, and once removed, the DW/Si-10 aerogel was capable of self-extinguishing. Both the silica aerogel and residual cellulose were intact; therefore, structural collapse was prevented. This simple and scalable wood enhancement method expands the application prospects of wood in the thermal insulation domain and provides a novel method for the exploitation and utilization of timber resources.

## 2. Results and Discussion

### 2.1. Microstructure and Composition

**Figure 2:**
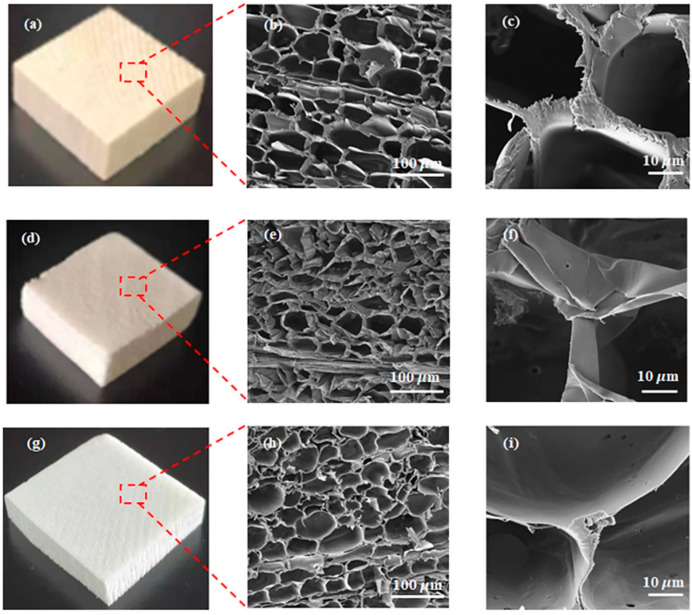
Optical photographs and SEM images of (**a**−**c**) NW, (**d**−**f**) DW, and (**g**−**i**) DW/Si-10 aerogel.

**Figure 3:**
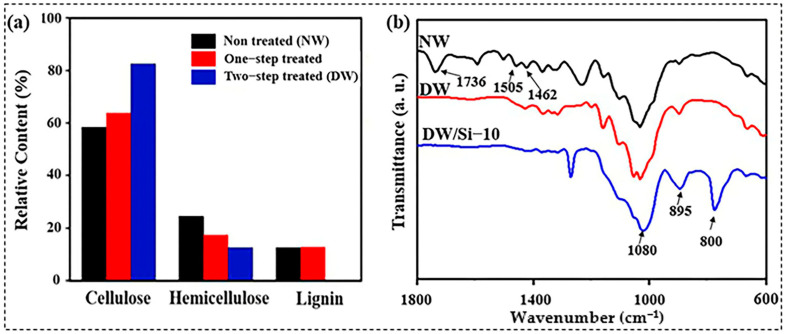
(**a**) Chemical composition and (**b**) FT−IR spectra of the samples.

Balsa wood has a density of approximately 94 mg cm^−3^. All lignin and partial hemicellulose were removed from the NW via a two-step chemical treatment, and wood-based aerogels were then prepared using the directional-freezing-assisted in situ sol–gel strategy. The cross-section of NW exhibited a honeycomb cell structure (Figure 2b,c), and its chemical composition primarily comprised cellulose, hemicellulose, and lignin [24]. As shown in Figure 3a, the contents of the three components in the NW were 58, 24, and 12%, respectively. Upon the completion of the two steps of chemical treatment, the lignin content in the wood decreased to 0%, thus evidencing that the lignin in the wood was completely removed, and only cellulose and a small amount of hemicellulose remained. As a result, the wood density decreased from 94 to 53 mg cm^−3^ due to the selective removal of hemicellulose and lignin (Table S1). Additionally, when comparing the physical appearance of the wood blocks before and after the chemical treatment, optical photographs (Figure 2a,d) revealed that the color of the wood became significantly lighter after the brown lignin was completely removed.

Further analysis with Fourier transform infrared (FT-IR) spectroscopy revealed that the characteristic peaks of lignin in DW at 1505 cm^−1^ and 1462 cm^−1^ (aromatic skeleton vibrations) and the hemicellulose-related peaks at 1736 cm^−1^ disappeared, indicating that the remaining components of wood primarily comprised cellulose after the two chemical treatments [25,26] (Figure 3b). Investigating the SEM images (Figure 2e,f) after the chemical treatment indicated that the cell wall of the wood became thinner; however, the wood multistage structure did not significantly change. This provided a structural basis for the subsequent silica nanoparticle modification. Owing to the removal of lignin and partial hemicellulose after the chemical reactions, void spaces were formed within the wood structure, thus generating binding sites for further in situ polymerization of silica particles.

**Figure 4:**
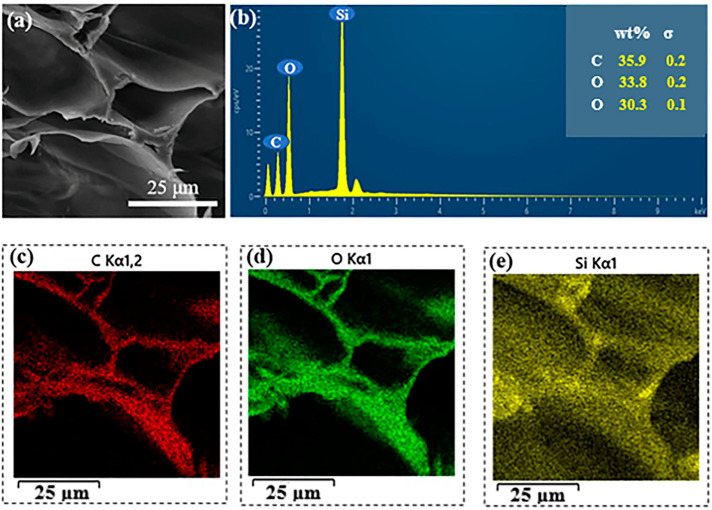
(**a**) SEM image of the DW/Si-10 aerogel, (**b**) Element (C, O, Si) content of the DW/Si-10 aerogel, (**c**) C, (**d**) O, and (**e**) Si EDS spectrum of the DW/Si-10 aerogel.

The prepared DW block was dipped into the prepared silica sol solution, where the sol solution rapidly entered the DW cell cavity due to the siphoning effect. The wet sample was then placed in a cold trap (−60 °C) for directional freezing. During the directional freezing of the sol solution, the forming ice crystals occupied the cell cavity, and the silica particles squeezed smoothly around and inside the cell wall, occupying the original position of the lignin by in situ polymerization. Energy-dispersive X-ray spectroscopy (EDS) analysis revealed the distribution of each element in the composite material. Figure 4a,b show that the C, O, and Si contents in the composite were 35.9, 33.8, and 30.3%, respectively. Figure 4c–e show that Si, O, and C were evenly distributed on the DW skeleton, thus demonstrating the uniform distribution of silica particles in the DW. In Figure 2h,i and Figure S1, the honeycomb-like cellular structure of the wood–silica aerogel is visible on the cross section, and a layer of tightly bonded silica particles are distributed on the cellulose skeleton. Homogeneity in a two-phase wood–silica composite can improve the hydrogen bond interactions between silica and cellulose molecules, which is favorable for the structural stability and performance of the composite.

The surface chemistry of the composite was confirmed using FT-IR spectroscopy (Figure 3b). In addition to the typical DW peaks, the peak at 1080 cm^−1^ was attributed to the asymmetric stretching vibration of Si-O-Si, and the characteristic peak at 800 cm^−1^ corresponded to the Si-O-Si symmetric stretching vibration, further stipulating and indicating that the silica aerogel was successfully grafted onto the surface of the wood skeleton [27,28]. In the case of silica aerogels, the characteristic peak of Si–OH was located at 920 cm^−1^, whereas the Si–OH characteristic peak of the wood–silica aerogels red-shifted to 895 cm^−1^. A red shift designates a decrease in the bond energy; thus, the results indicate that Si–OH generated hydrogen bonds with the −OH groups on the surface of the wood skeleton. Figure S2 shows the X-ray diffraction (XRD) patterns of the NW, DW, and DW/Si-10 aerogels. The XRD patterns exhibited characteristic diffraction peaks of (101), (002), and (040) at 2*θ* = 17°, 22.5°, and 35°, respectively [29]. With the introduction of silica aerogel, the positions of the wood cellulose diffraction peaks were invariable, which shows that the cellulose crystalline structure was not destroyed in the composite. However, the intensity of the (040) cellulose diffraction peak decreased significantly because of partial coverage. In addition, the broad amorphous peak of silica at 22.5° increased significantly, confirming the presence of silica nanoparticles on the wood skeleton surface.

The directional-freezing-assisted in situ gel strategy uniformly wrapped the high-strength silica sol–gel particle network on the cellulose surface, and the two-phase composite system was more homogeneous. Strong hydrogen bond interactions between the silica and cellulose enabled the two phases to achieve molecular-level recombination, reaching a more stable combination. Therefore, the composite exhibits superior mechanical strength, hydrophobicity, fire resistance, and heat insulation properties in comparison to natural wood. The modified material demonstrated in this study stipulates a great potential for development in the thermal insulation industry worldwide.

### 2.2. Mechanical Properties

**Figure 5:**
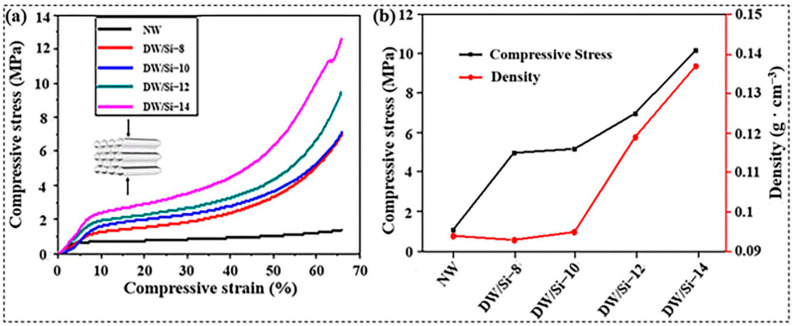
(**a**) Stress−strain curves of the samples (inset is the schematic of compression test) and (**b**) elastic modulus and strength at 60% compression of the NW and DW/Si-x aerogels.

Stress–strain curves were used to characterize the radial compressive strength of the wood samples in order to investigate the mechanical properties of the materials (Figure 5). The mechanical behavior of wood under radial compression primarily comprised three stages: linear elasticity, long strain yield, and densification [30,31]. The elasticity of the materials, *ε*, of <10% in the linear elastic stage reflects the bending of cell walls in the samples. When *ε* increased to 40%, the strain yield stage was long. Owing to the gradual compression of the layered wood structure, a stress plateau region was observed. When *ε* was >40%, the rapid increase in the densification stage was caused by the continuous deformation of the layered structure. The results indicate that at a strain of 60%, the radial compressive strength of NW was 1.1 MPa. However, when the lignin and partial hemicellulose were removed, the radial compressive strength of the wood decreased to 0.31 MPa (Figure S3). The removal of lignin and hemicellulose decreased the wood density; therefore, more voids were exposed, which significantly weakened the mechanical properties of the wood. However, the removal of lignin and hemicellulose created more vacancies for the introduction of high-strength silica particles, which replaced lignin and formed strong hydrogen bonds with ordered cellulose, the improvement of the two phases’ interface interactions imbuing the composite wood with excellent mechanical properties. Table S1 illustrates that, as the amount of silica content increases, the radial compressive strength of the wood composite increases up to 10 MPa (60% strain). The density of DW-Si-10 is similar to that of NW; however, its strength was up to 5.2 MPa, which is approximately five times that of NW. In addition, the comparison test of tensile strength between the natural wood (NW) and the as-prepared composite DW/Si-10 was carried out. As shown in Figure S5, the result shows that the tensile strength of the composite DW/Si-10 (0.76 MPa) is significantly better than that of NW (0.49 MPa). The outstanding mechanical properties of the composite wood were attributed to the uniform combination of high-strength silica particles and arranged cellulose. The directionally arranged cellulose nanofibers and silica achieved molecular-level fusion, thus forming strong hydrogen-bond interactions between the two phases, which significantly improved the mechanical properties of the composite wood. The outstanding mechanical properties of wood are essential in ensuring its comprehensive utilization.

### 2.3. Thermal Insulation

**Figure 6:**
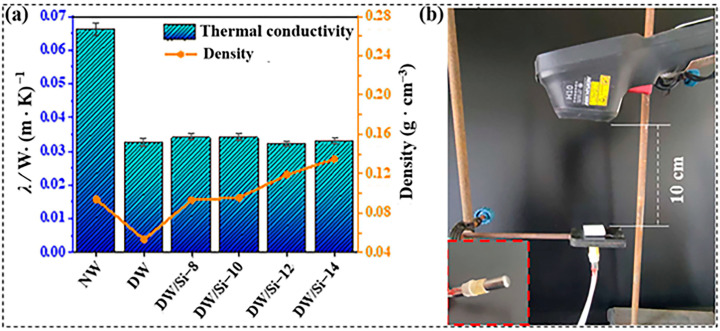
(**a**) Densities and thermal conductivity of the samples and (**b**) the experimental setup; inset is the point heat source.

The thermal conductivity coefficient, a basic parameter of thermal transmission theory, is one of the most important indices for evaluating the thermal insulation performance of porous materials. The internal heat conduction of porous materials includes three main modes: thermal radiation, air heat conduction, and solid heat conduction [32]. It is noted that the effect of thermal radiation at room temperature is negligible. Solid heat conduction depends primarily on the inherent properties of the material and air heat conduction depends on the microstructure of the material. For equivalent materials, as the density decreases, the solid heat conductivity decreases. When the pore size of highly porous materials is smaller than the molecular free path of air (approximately 70 nm), air heat conduction is limited. After the two-step chemical treatment, where lignin and cellulose were removed from the NW, the thermal conductivity coefficient of wood decreased from 0.066 to 0.032 W (m·K)^−1^ (Figure 6a). It was noted that the main factor affecting the change in the thermal conductivity of the DW was the decrease in wood density, this resulting in an impeded heat conduction. Notably, the density of the DW/Si-10 composite aerogel was similar to that of NW, but its thermal conductivity was only half that of NW. This is because silica aerogel is a typical mesoporous material with low density and high porosity [19,33]. After the silica particles substituted and occupied the original position of lignin, solid and air heat conduction were both limited simultaneously; thus, they exhibited a lower thermal conductivity coefficient and better thermal insulation performance in comparison to NW. In addition, the thermal conductivity of DW/Si-10 aerogel was compared with other wood-based composites reported in recent years, The achieved thermal conductivity value of the DW/Si-10 composite aerogel (0.034 W (m·K)^−1^) was lower than the thermal conductivity of that of other wood-based composites, such as thermal energy storage wood (TESW) (0.098 W (m·K)^−1^) [34], nanofibril network filling wood aerogel (0.057 W (m·K)^−1^) [8], silica-aerogel-impregnated wood (S-40-5) (0.0.068 W (m·K)^−1^) [22], and concrete–coconut (0.170 W (m·K)^−1^) [23], as shown in Table S2.

**Figure 7:**
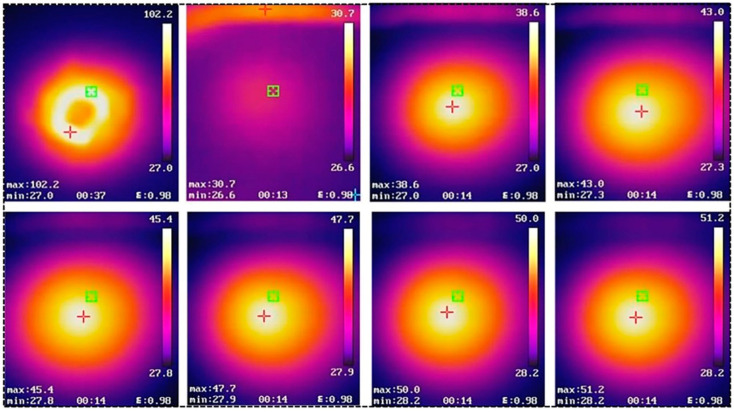
Infrared images of the DW/Si-10 aerogel at a point heat source of 102 °C; “max” and “min” in the Figure body express the highest and minimum temperature in the infrared region, respectively.

An infrared thermal imager was used to investigate the thermal insulation properties of the composite materials (Figure 6b). The sample was placed directly above the point heat source, and the two were in contact. The bottom of the sample was heated, and the front temperature of the sample was recorded every 10 s. After 60 s, the front surface temperature of NW increased to 57.9 °C (Figure S4), whereas that of the DW/Si-10 increased only to 51.2 °C (Figure 7). A comparison of the temperature changes of the NW and DW/Si-10 aerogels further confirmed that the wood-based aerogel possessed better thermal insulation properties than NW.

### 2.4. Hydrophobicity

**Figure 8:**
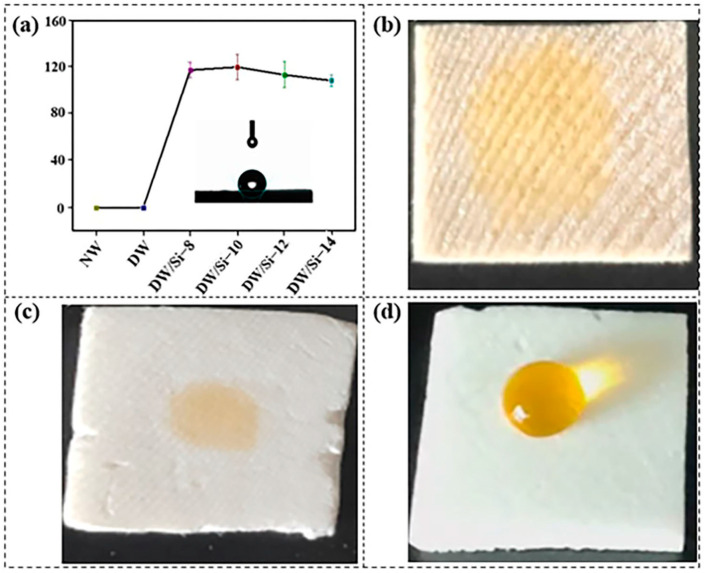
(**a**) WCAs of the samples. Water dropped onto the surface of (**b**) NW, (**c**) DW, and (**d**) DW/Si-10; the water was colored orange with methyl orange before the experiments; inset is the morphology of water droplets on the DW/Si-x material surface.

The hydrophobic properties of NW, DW, and DW/Si-10 were investigated by measuring the water contact angles (WCAs) at room temperature. As shown in Figure 8a, the results indicate that the WCAs of the NW and DW were both equal to 0°. In contrast, the DW/Si-10 aerogel exhibited a contact angle of up to 120°, indicating very high hydrophobicity. For further investigations, water droplets were deposited on the NW, DW, and DW/Si-10 skeleton surfaces; NW and DW exhibited significant hydrophilic properties where water was instantaneously absorbed (Figure 8b,c). On the contrary, water remained on the surface of the DW/Si-10 aerogel, thus exhibiting lacking affinity for water and indicating high hydrophobicity (Figure 8d). Videos S1–S3 displays the significant variation of hydrophobic properties between the samples.

The wettability of a material depends on both the chemical composition and geometrical microstructure of the solid interface based on Wenzel’s theory [35,36,37]. According to the thermodynamic equilibrium, the apparent contact angle and roughness factor possess the following relationship.

(1) $$\cos\theta^{w}=r\cos\theta$$

where *θ^w^* denotes the apparent contact angle; *r* is the roughness factor of the given interface; and *θ* is Young’s angle. Herein, several hydrophobic groups (−CH_3_) were exposed on the material surface, the surface energy of the wood was effectively decreased by the hydrophobic groups (−CH_3_), and the silica nanoparticles increased the surface roughness of the wood. Therefore, the composite exhibited excellent hydrophobicity. In conclusion, the excellent hydrophobicity of the wood-based composite is beneficial to a prominent thermal insulation performance in a high-humidity environment.

### 2.5. Thermal Stability and Fire Resistance

**Figure 9:**
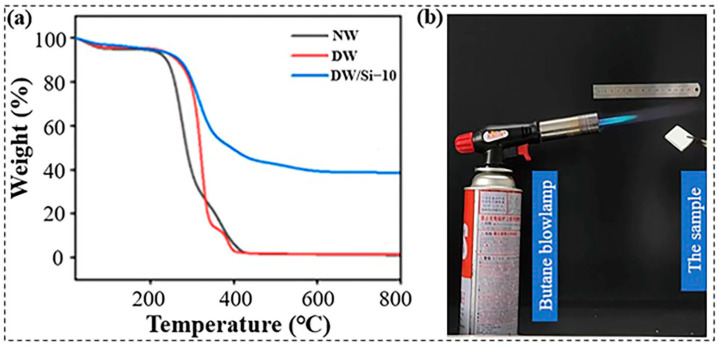
(**a**) TGA curves (measured in air), and (**b**) the experimental setup.

Thermal stability and fire resistance are important properties for the safety of wood. First, the thermal stabilities of NW, DW and DW/Si-10 were investigated using thermogravimetric analysis (TGA) (Figure 9a). The TGA curves indicate that the decomposition temperature of wood gradually increased from 276 to approximately 320 °C, thus confirming that the thermal stability of the wood improved by approximately 45 °C after lignin removal, which indicates that DW can be used at higher temperatures. With the introduction of silica aerogel, the complete degradation temperature of the composite increased from 400 °C to >600 °C, and 38% of the remaining components were retained. This shows that the composite was capable of maintaining its structural integrity after burning. To evaluate the fire resistance of the DW/Si-10 aerogel, the variable time upon which the composite aerogel was subjected to the flame of a butane blowlamp (approximately 1300 °C) was recorded and studied (Figure 9b). The sample was removed from the flame of a butane blowlamp after exposure for 5 s and its combustion was observed. DW continued to combust until the material was completely burned into ashes (Video S4). Notably, the DW/Si-10 aerogel self-extinguished after being removed from the direct flame of the blowlamp. Both the silica aerogel and residual cellulose were intact, and structural collapse was prevented (Video S5).

The results indicate that the addition of silica aerogel not only delayed the ignition time of wood but also generated a significant self-extinguishing effect. Moreover, the structural integrity of the composite was maintained after combustion. The exceptional fire resistance of the composite was attributed to the inorganic silica particles attached to the cellulose, forming a fire-resistant protective layer, which decreased the rate of combustion by limiting thermal diffusion.

Furthermore, the fire-resistant DW/Si-10 aerogel was tested against other commercial thermal insulation materials such as polyurethane foam and polyethylene benzene foam. Polyurethane foam ignited rapidly and released a large amount of smoke when encountering a butane flame (Video S6); similarly, polyethylene benzene foam also ignited completely without leaving any ashes (Video S7). The fire resistance of these two commercial materials was inferior to that of the composite aerogels, emphasizing the significant potential advantages of our modified composite material in the thermal insulation industry.

## 3. Conclusions

A directional-freezing-assisted in situ sol–gel approach was proposed to create wood–silica composite aerogels with enhanced mechanical, fire-resistant, and hydrophobic properties. The forming ice crystals during the directional freezing process successfully squeeze silica nanoparticles around and inside the cell wall of wood, enabling silica nanoparticles to successfully replace lignin and achieve a combination with cellulose at the near-molecular level, and enable the formation of strong hydrogen bonds between the silica and cellulose molecules, which imbues the wood-based composite aerogels with a series of outstanding properties: (1) The prepared DW/Si-10 sample exhibited a density similar to that of NW and a radial compressive strength significantly higher than that of NW as well as (2) a thermal conductivity coefficient that was half that of NW; (3) The enhanced aerogel composite possesses high hydrophobicity, which is beneficial to a prominent thermal insulation performance in a high-humidity environment; (4) Self-extinguishing properties were observed when DW/Si-10 was ignited by a direct flame, where the complete structure was intact after the combustion, thus demonstrating outstanding fire resistance. The application of this simple yet advanced technique to process raw wood will result in a highly compatible advanced insulation material possessing high strength and outstanding fire-resistant and hydrophobic characteristics. The developed composite material does not only improve energy utilization efficiency and long-term service stability but also provides a reference for improving the safety of thermal insulation materials.

## 4. Materials and Methods

### 4.1. Materials and Chemicals

Balsa wood with a density of ~94 mg cm^−3^ was used to fabricate the wood-based composite aerogels. The wood samples were cut to the dimensions of 30 × 30 × 5 mm^3^ (radial × tangential × longitudinal). The reagents, including methyltriethoxysilane (MTES, ≥98%), sodium chlorite (NaClO_2_, 80%), acetic acid (AR), hydrochloric acid (HCl, AR), sodium sulfite (Na_2_SO_3_, AR), and sodium hydroxide (NaOH, AR) were bought from Shanghai Macklin Biochemical Co., Ltd. (Shanghai, China). All of these chemicals were used as received without further purification. The de-ionized water was used to make up all mixed solutions and throughout the experiments.

### 4.2. Preparation of Wood-Based Aerogel

Firstly, the lignin and hemicellulose fractions were selectively removed from the cell wall of the wood sample, followed by freeze-drying. Specifically, the natural wood samples were immersed in a mixture solution (2.5 M NaOH and 0.4 M Na_2_SO_3_) at 100 °C for 10 h, followed by washing several times in deionized water to remove the chemicals. Then, the samples were initially delignified using an aqueous solution of 2 wt% NaClO_2_ buffered with acetic acid at pH 4.7 at 100 °C for 3 h. The treated samples were carefully washed with deionized water to remove the remaining chemicals. Finally, the samples were freeze-dried for over 24 h, resulting in the formation of delignified wood (DW). A facile directional-freezing-assisted in situ sol–gel strategy was carried to produce the composite aerogels. Firstly, a certain amount of MTES and deionized water (22.4 mL) were added into a beaker successively under even stirring. After 0.2 mL of hydrochloric acid (*w*/*w* 1% HCl) was added into the beaker, stirring was performed for 0.5 h. After that, a certain amount of NaOH (1 mol/L) was added rapidly. Then, the re-prepared DW samples soaked in the silica solution above mentioned. Then, the wet samples were pulled out of the liquid phase and moved to the cold trap (−60 °C). Then, the hydrogel was frozen via the directional freezing method, followed by freeze-drying for over 24 h to obtain the composite aerogel DW/Si-x. For the different amounts of MTES, the wood-based composite aerogels were named as follows: DW/Si-8, DW/Si-10, DW/Si-12, and DW/Si-14, recorded in Table 1.

### 4.3. Characterization

The morphology and structure of the wood samples were characterized via field-emission scanning electron microscopy (SEM, Thermo Scientific Apreo 2C, Thermo Scientific, Waltham, MA, USA) with an energy-dispersive X-ray spectroscopic detector (OXFORD ULTIM Max65, Britain) for mapping. FT-IR spectra were recorded on a Thermo Scientific Nicolet iS10 (Thermo Scientific, Waltham, MA, USA) spectrometer (America). All spectra were recorded between 1800 and 600 cm^−1^ with 4 cm^−1^ and 16 scans per sample. The crystal structures were measured using an Ultima IV X-ray diffraction-meter in the range of 5–40° (2*θ*). The mechanical compressibility of the samples was measured using a universal testing machine (WDW-D1000N, Jinan Xinguang Testing Machine Manufacturing Co., Ltd., Shandong, China) when all the samples were cut into regular cuboid form (10 × 5 × 5 mm). The strain ramp rate was confined to 5 mm/min for the tests. The thermal conductivity of the samples was evaluated using a C-Therm TCi thermal conductivity analyzer (Fredericton, NB, Canada) through a transient plate method. The thermal degradation behavior of the samples in air was investigated using thermogravimetry analysis (TGA5500, USA). The sample was placed in a ceramic pan and heated from 20 to 800 °C at a heating rate of 10 °C/min. Static contact angles of the samples were performed using a contact angle measuring system (OCA 50, Dataphysics, Stuttgart, Germany) at room temperature. For each sample, at least three specimens were tested, and the average value was reported. Infrared imaging was performed using a thermal infrared camera (H10, HIKMICRO). The camera was operated at a distance of approximately 10 cm. The porosity of DW/Si-x aerogels was calculated according to Equation (S1) [38,39]. Acid-insoluble lignin content in the samples was determined with a 72 wt% sulfuric acid solution according to the GB/T 20805-2006 standard; holocellulose and α-cellulose contents were measured in accordance with the GB/T 20806-2006 standards, respectively. The water contact angles (WCAs) and the thermal conductivity coefficient test of all samples were repeated three times.

## Figures and Tables

**Table 1:** The amount of silica source (MTES) in the samples.

Samples	NW	DW	DW/Si-8	DW/Si-10	DW/Si-12	DW/Si-14
MTES (mL)	0	0	8	10	12	14

## Data Availability

The data presented in this study are available on request from the corresponding author.

## Supplementary Materials

The following supporting information can be downloaded at: https://www.mdpi.com/article/10.3390/gels9020170/s1, Video S1: Video of the process of hydrophobicity test of NW; Video S2: Video of the process of hydrophobicity test of DW; Video S3: Video of the process of hydrophobicity test of DW/Si-10 aerogel; Video S4: Video of the combustion process of NW; Video S5: Video of the combustion process of DW/Si-10 aerogel; Video S6: Video of the combustion process of polyurethane foam; Video S7: Video of the combustion process of polyethylene benzene foam; Figure S1: SEM image of DW/Si-10 aerogel; Figure S2: XRD patterns of NW, DW, and DW/Si-10 aerogel; Figure S3: Stress-strain curves of DW; Figure S4: Infrared images of NW; Figure S5: The tensile stress–strain curves of NW and DW/Si-10 aerogel. Table S1: The physical properties of the samples. Table S2: Comparison of thermal conductivity of the DW/Si-10 composite aerogel with some other wood-based composites used for thermal insulation.
