# Supplementary material for: Directional-Freezing-Assisted In Situ Sol–Gel Strategy to Synthesize High-Strength, Fire-Resistant, and Hydrophobic Wood-Based Composite Aerogels for Thermal Insulation
Source: Gels. 2023 Feb 20;9(2):170. doi: 10.3390/gels9020170 (PMC9956576; doi:10.3390/gels9020170)
Supplement: Supplementary file 1 [file gels-09-00170-s001.zip › gels-2210827-supplementary-for proof-Revised.pdf]

gels

Supplementary Materials

Directional-Freezing-Assisted In Situ Sol–gel Strategy to Synthesize High-Strength, Fire-Resistant, and Hydrophobic Wood-Based Composite Aerogels for Thermal Insulation

Yan Hou †, Junyong Chen *,†, Defang Pan and Lu Zhao

This supplementary information contains:
1. Supplementary movies
2. SEM image of DW/Si-10 aerogel
3. XRD patterns of NW, DW, and DW/Si-10 aerogel
4. Stress–strain curves of DW
5. Infrared images of NW
6. Tensile stress–strain curves of NW and DW/Si-10 aerogel
7. Physical properties of the samples
8. Comparison of thermal conductivity of the different wood-based composites

1. Supplementary Movies

Movie S1

Movie of the process of hydrophobicity test of NW.

Movie S2

Movie of the process of hydrophobicity test of DW.

Movie S3

Movie of the process of hydrophobicity test of DW/Si-10 aerogel.

From these movies S1~S3, it was found that the newly developed composite overcomes the strong hydrophilicity of the wood materials (NW and DW) and the water resistance was significantly improved. The difference of hydrophobicity between the composite and the wood samples is caused by their obvious distinction in microstructure and surface chemistry. Hydrophobic groups (–CH_3) were introduced onto the wood surface, the surface energy of the wood was effectively decreased by the hydrophobic groups, and the silica nanoparticles increased the surface roughness of the wood. Therefore, the composite exhibited excellent hydrophobicity. The excellent hydrophobicity of the wood is conducive to maintaining thermal insulation performance in a high-humidity environment.

Movie S4

Movie of the combustion process of DW.

Movie S5

Movie of the combustion process of DW/Si-10 aerogel.

Movie S6

Movie of the combustion process of polyurethane foam.

Movie S7

Movie of the combustion process of polyethylene benzene foam.

2. SEM of DW/Si-10 aerogel

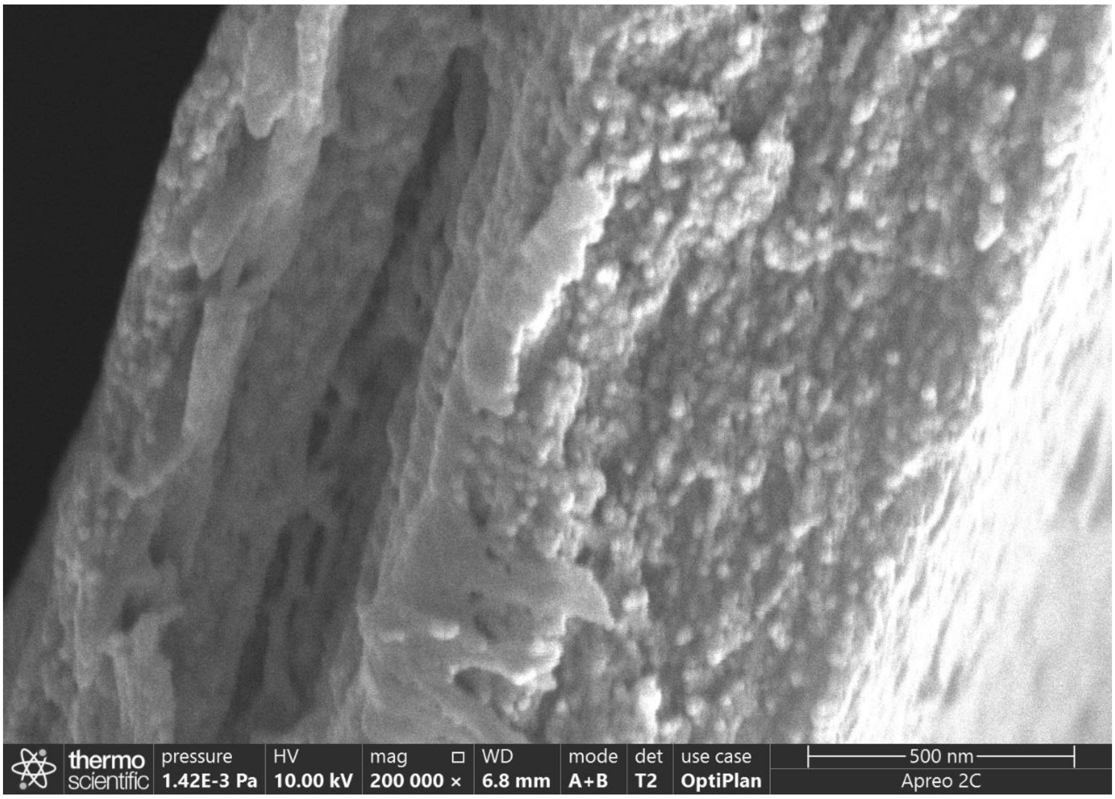

Figure S1. SEM image of DW/Si-10 aerogel.

3. XRD patterns of NW, DW, and DW/Si-10 aerogel

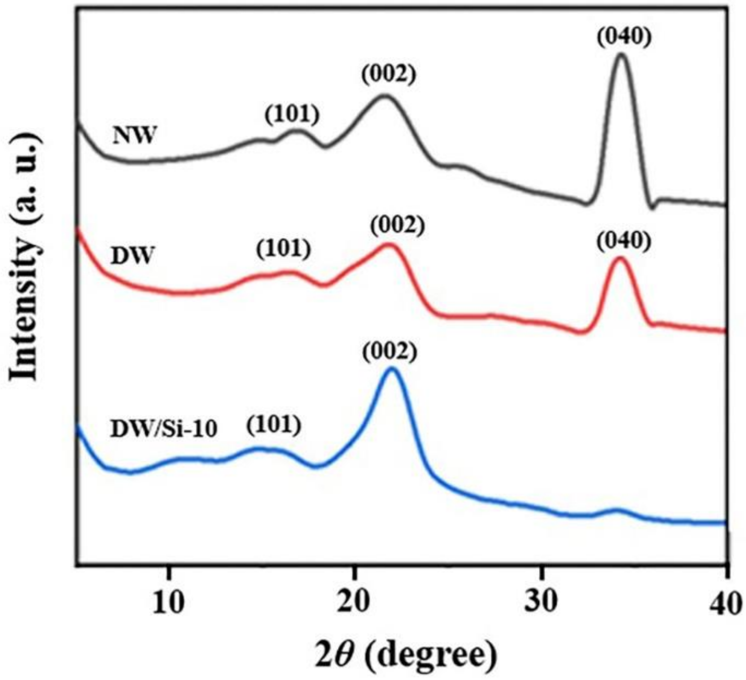

Figure S2. XRD patterns of NW, DW, and DW/Si-10 aerogel.

4. Stress-strain curves of DW

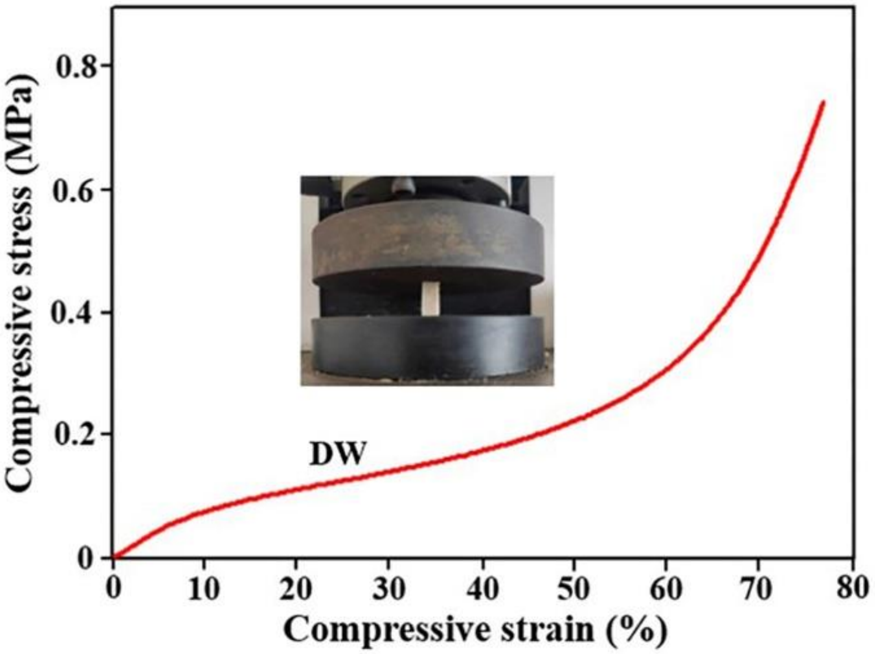

Figure S3. Stress–strain curves of DW.

5. Infrared images of NW

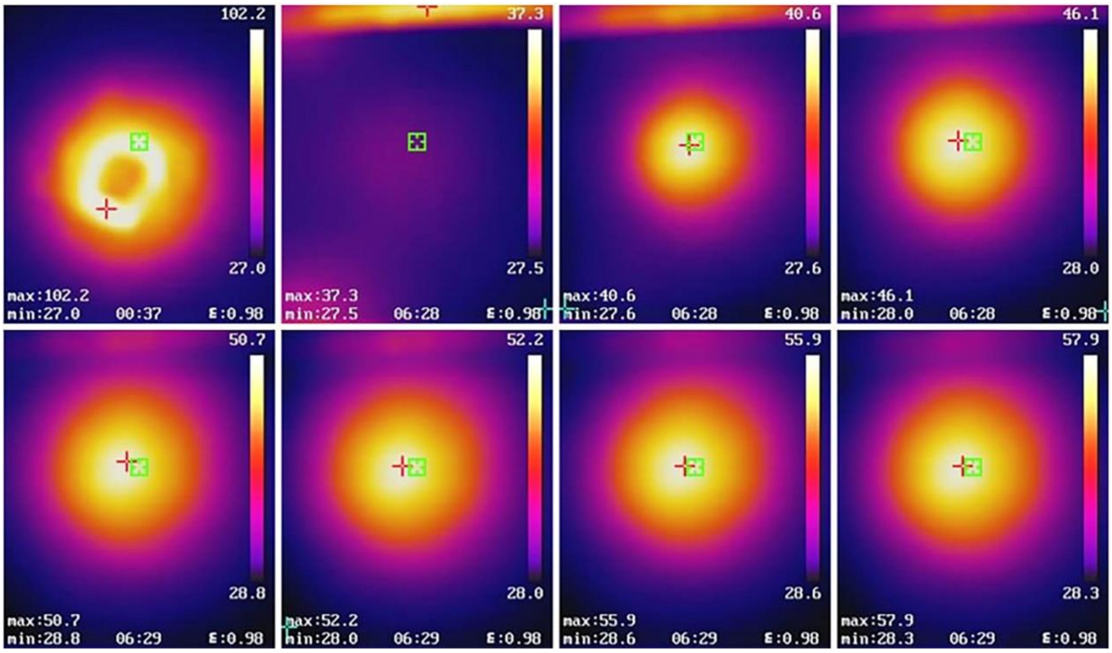

Figure S4. Infrared images of NW within a minute of being exposed to a point heat source at 102 °C.

6. Tensile stress–strain curves of NW and DW/Si-10 aerogel

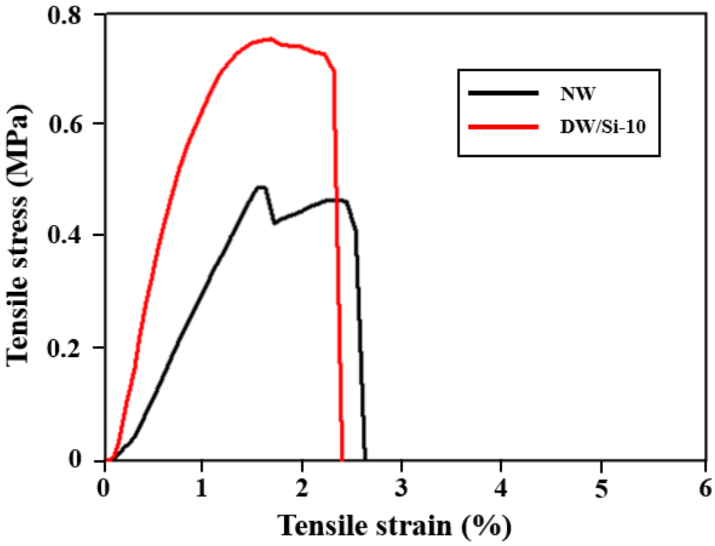

Figure S5. The tensile stress–strain curves of NW and DW/Si-10 aerogel.

7. Physical properties of the samples

Table S1. The properties of NW, DW and the composites with different silica content.

Samples	Density (g cm^{-3})	Thermal conductivity (W (m K)$^{-1}$)	Porosity (%)	Compressive strength (MPa)
NW	0.094	0.066	93.7	1.1
DW	0.053	0.032	96.5	0.31
DW/Si-8	0.093	0.034	94.7	5
DW/Si-10	0.096	0.032	94.6	5.2
DW/Si-12	0.119	0.032	93.5	7
DW/Si-12	0.137	0.033	92.7	10.2

The apparent volumetric mass density of the samples was calculated by measuring the weight and volume of each individual sample. The porosity of DW/Si-x aerogels was calculated according to eq 1, where ϱ, $ϱ_s$ and $ϱ_c$ are the bulk density of DW/Si-x aerogels and the skeleton densities of pure silica aerogels and cellulose; ω_s and ω_c are the mass fraction of silica and cellulose in the DW/Si-x aerogels, respectively. Herein, based on literature data, $ϱ_s$ and $ϱ_c$ were designed as 2.1 g cm^{-3} and 1.5 g cm^{-3} [38,39].

$$Porosity\ (\%) = \left(1 - \frac{\rho}{\omega_s\rho_s + \omega_c\rho_c}\right) \times 100\% \quad (1)$$

8. Comparison of thermal conductivity of the different wood-based composites

Table S2. Comparison of thermal conductivity of the DW/Si-10 composite aerogel with some other wood-based composites used for thermal insulation.

Composite	K (W (m·K)$^{-1}$)	Ref.
Thermal energy storage wood (TESW)	0.098	[34]
Nanofibril network filling wood aerogel	0.057	[8]
Silica-aerogel-impregnated wood (S-40-5)	~0.06	[22]
Wood–silica aerogels	0.068	[23]
DW/Si-10	0.034	This work

Reference

38. Wang, Z.X.; Han, X.S.; Han, X.W.; Chen, Z.B.; Wang, S.J.; Pu, J.W. MXene/wood-derived hierarchical cellulose scaffold composite with superior electromagnetic shielding. *Carbohyd. Polym.* **2021**, 254, 117033–117041.

39. Sai, H.Z.; Fu, R.; Xiang, J.H.; Guan, Y.L.; Zhang, F.S. Fabrication of elastic silica-bacterial cellulose composite aerogels with nanoscale interpenetrating network by ultrafast evaporative drying. *Compos. Sci. Technol.* **2017**, 155, 72–80.
34. Lin, X.X.; Jia, S.F.; Liu, J.Y.; Wang, W.B.; Cao, H.M.; Guo, X.; Sun, W.S. Fabrication of thermal energy storage wood based on graphene aerogel encapsulated polyethylene glycol as phase change material. *Mater. Res. Express* **2020**, 7, 95503–95512.
8. Garemark, J.; Perea-Buceta, J.E.; Del Cerro, D.R.; Hall, S.; Berke, B.; Kilpelainen, I.; Berglund, L.A.; Li, Y.Y. Nanostructurally Controllable Strong Wood Aerogel toward Efficient Thermal Insulation. *Acs Appl. Mater. Inter.* **2022**, 14, 24697–24707.
22. Chen, H.; Zhang, Y.T.; Zhong, T.H.; Wu, Z.H.; Zhan, X.X.; Ye, J.Y. Thermal insulation and hydrophobization of wood impregnated with silica aerogel powder. *J. Wood Sci.* **2020**, 66, 81–91.
23. Yan, M.Y.; Fu, Y.Y.; Pan, Y.L.; Cheng, X.D.; Gong, L.L.; Zhou, Y.; Ahmed, H.; Zhang, H.P. Highly elastic and fatigue resistant wood/silica composite aerogel operated at extremely low temperature. *Compos. Part B-Eng.* **2022**, 230, 109496–109506.
