# Supplementary material for: Directional-Freezing-Assisted In Situ Sol–Gel Strategy to Synthesize High-Strength, Fire-Resistant, and Hydrophobic Wood-Based Composite Aerogels for Thermal Insulation
Source: Gels. 2023 Feb 20;9(2):170. doi: 10.3390/gels9020170 (PMC9956576; doi:10.3390/gels9020170)
Supplement: Supplementary file 1 [file gels-09-00170-s001.zip › Videos S1-S7/name.pdf]

2. The Hydrophobicity Test of DW

3. The Hydrophobicity Test of DW/Si-10

4. The Combustion Process of NW

5. The Combustion Process of DW/Si-10

6 The Combustion Process of Polyurethane Foam

7 The Combustion Process of Polyethylene Benzene Foam
